# Editorial: Mechanotransduction in vascular development and disease

**DOI:** 10.3389/fphys.2024.1525184

**Published:** 2024-12-10

**Authors:** Nicolas Baeyens, Brian G. Coon, Julia J. Mack

**Affiliations:** ^1^ Laboratoire de Physiologie et Pharmacologie, Faculté de Médecine, Université libre de Bruxelles, Brussels, Belgium; ^2^ Cardiovascular Biology Program, Oklahoma Medical Research Foundation, Oklahoma City, OK, United States; ^3^ Department of Cell Biology, University of Oklahoma Health Sciences Center, Oklahoma City, OK, United States; ^4^ Department of Medicine, Division of Cardiology, University of California, Los Angeles, Los Angeles, CA, United States

**Keywords:** blood flow, hemodynamics, shear stress, vascular disease, mechanotransduction, vascular development, endothelial

This Research Topic of reviews and research articles focuses on the Research Topic of vascular mechanotransduction. Articles cover various aspects of vascular mechanobiology, but with special emphasis on understanding the role of the hemodynamic microenvironment on cell signaling, cellular organization, vascular inflammation, and physiological function.

Endothelial cell responses to blood flow depends on various environmental and intrinsic factors. However, a typical response involves cytoskeletal, nuclear, and Golgi apparatus orientation in line with the flow direction. Through a thorough *in vivo* experiment, Nicolas et al. accurately defined the hemodynamics parameters of the mouse carotid artery during systole and diastole, estimating wall shear stress by using a modified equation to estimate blood behavior in smaller vessels. They analyzed endothelial cell polarity by *en-face* staining, confirming that endothelial cells align against flow direction in small arteries. Continuing in the theme of endothelial cell polarity with flow, *in vitro* work from Moise et al. deciphered how physiological and unidirectional wall shear stress transduces endothelial cell polarization through acetylation of microtubules and increased polymerization, bridging flow-mediated cell polarity with all components of the cellular cytoskeleton.

Hemodynamic forces are not uniform through the vascular network and organotypic adaptation is a critical feature of organ function. Thus, there is increasing interest to model hemodynamics and vessel structure/function in organoids. The review by Martier et al. summarizes current platforms and discusses *in vitro* models that include a vascular compartment. Considering mechanosensitive signaling regulates the characteristics of a healthy vasculature, *in vitro* models that include hemodynamic flow will better recapitulate *in vivo* settings. In one example, Hong et al. use an *in vitro* system to recapitulate the different endothelial cell phenotypes associated with unidirectional laminar flow (UF) and disturbed flow (DF). Performing complementary RNA sequencing and lipidomics, the authors revealed that genes associated with lipid metabolism and specific lipid species are altered under DF. Interestingly, they found that the endothelial inflammatory state, induced by DF or an inflammatory agonist, increases total lipid abundance in cells. Whether this lipid increase is a protective response of the cell is unknown. Ultimately, the work provides transcriptomic and lipidomic datasets of aortic endothelial cells to interrogate how the local flow pattern contributes to vascular inflammation.

Regarding fundamental cell responses to shear stress, Bougaran and Bautch review the roles of the nuclear LINC complex in endothelial mechanotransduction and force sensing. The review touches on key functions of the LINC complex components, including SUN and nesprin proteins. It describes recent evidence that SUN1 and SUN2 orchestrate a mechanosensing response that extends both inward to the nuclear chromatin and outward to cell-cell and cell-matrix junctions. The authors further discuss these findings with vascular pathologies such as Hutchinson-Gilford progeria syndrome (HGPS), a premature aging disorder with cardiovascular impairment. Following the importance of nuclear mechanosensing, Shores and Truskey review HGPS disease pathology and the disrupted mechanics of nuclear mechanotransduction. The disease, associated with the accumulation of progerin, a mutated form of the nuclear lamina protein lamin A, disrupts nuclear integrity and leads to cell senescence and overall dysfunction. The review focuses on the consequence of progerin accumulation in vascular cells, specifically endothelial and smooth muscle, and suggests that dysfunctional mechanotransduction plays a role in the pathobiology of HGPS.

The adventitia of the aorta is lined with connective tissue, fat, lymphatics, and microvasculature. As aortic segments have different developmental origins and propensity to disease, it is important to consider the local vessel composition. The study by Rendon et al. focuses on the spatial-temporal changes to adipocyte progenitor cell (APC) and APC-subtype populations along the aortic and mesenteric vascular systems. Using short-term labeling of APCs, the authors found that aged mice lose perivascular APCs in the thoracic aortas but gain them, preferentially in female mice, in the mesenteric vasculature without a significant change in blood pressure. Notably, the adventitia of the thoracic and abdominal aorta is associated with different expression of APC subtype markers.

Moving onto the cerebral vasculature, the review by Dmytriv et al. discusses the onset and progression of cognitive impairment associated with chronic vascular encephalopathy (CVE). CVE occurs after prolonged reduction of blood flow to the brain. The lack of cerebral blood flow causes insufficient oxygenation, leading to hypoxia and the development of oxidative/reductive stresses. The authors discuss how the hypoxia-induced challenges in CVE, specifically oxidative stress and inflammation, work in concert to advance degeneration. Interestingly, they highlight potential therapeutic approaches to treating CVE, to alleviate the cognitive impairments.

Considering the global population is aging along with an increase in aging-associated diseases such as Alzheimer’s disease (AD), O’Hare et al. elaborate on their hypothesis that AD is linked to dysfunctional endothelial glycocalyx. The glycocalyx is a network of plasma membrane glycoproteins and proteoglycans that act as a structural and chemical barrier. In many vascular pathologies, such as sepsis, the glycocalyx is shed concurrently with disrupted cell function. There are also instances whereby the glycocalyx thins, which might be associated with endothelial impairment. The authors review the roles of the glycocalyx and discuss the importance of perfectly tuned endothelial function in neurovascular coupling. As neurovascular coupling defects are likely major contributors to disease progression, the authors make a strong case for considering endothelial drivers of AD.

Finally, maternal vascular adaptation during pregnancy is critical for the developing fetus. For the treatment of medical complications of pregnancy, we need a better understanding of vascular remodeling during embryogenesis. Unfortunately, research in this area is complicated because human samples are difficult to obtain, and there are differences between human and mouse extraembryonic vascular structures. Van Schoor et al. review the development of the extra-embryonic circulatory systems of both mice and humans, the associated hemodynamics, and mechanosensitive signaling pathways likely to guide vascular remodeling. Since proper vascular network development involves blood flow-independent and blood flow-dependent pathways, further mechanistic studies are needed. The review brings insights into the role of hemodynamics as a cause or consequence of extraembryonic vascular remodeling.

In summary, the role of blood flow forces and cell mechanotransduction must be addressed in the context of vascular health. This Research Topic highlights the consequences of effective and defective mechanosensitive signaling and reminds us that healthy vessels are associated with healthy blood flow.

